# FDA-Approved Passive Immunization Treatments Against Aβ in Alzheimer’s Disease: Where Are We Now?

**DOI:** 10.3390/ijms27020883

**Published:** 2026-01-15

**Authors:** Martin Higgins, Veronica Wasef, Andrea Kwakowsky

**Affiliations:** Pharmacology and Therapeutics, School of Pharmacy and Medical Sciences, Institute for Health Discovery and Innovation, Institute for Clinical Trials, Galway Neuroscience Centre, University of Galway, H91 TK33 Galway, Ireland; martyhiggins7@gmail.com (M.H.); v.wasef1@universityofgalway.ie (V.W.)

**Keywords:** Alzheimer’s disease, *APOE4*, ARIA, mAb, Aβ

## Abstract

Alzheimer’s disease (AD) is a progressive neurodegenerative disorder marked by decreased amyloid-beta (Aβ) clearance, enhanced Aβ aggregation, an increased risk of amyloid-related imaging abnormalities (ARIA), and blood–brain barrier (BBB) dysfunction. The *APOE4* allele, being the leading genetic risk factor for AD, contributes strongly to these symptoms. This review covers the relationship between *APOE4* status and the efficacy of FDA-approved monoclonal antibody (mAb) therapies, namely aducanumab, lecanemab, and donanemab. Across several clinical trials, *APOE4* carriers exhibited higher rates of ARIA-E and ARIA-H compared to non-carriers. While the therapies did often meet biomarker endpoints (i.e., reduced amyloid), benefits were only observed in early and mild AD, and cognitive benefits were often marginal. Going forward, experimental apoE4-targeted immunotherapies may ease the burden of *APOE4*-related pathology. The field is shifting towards a more integrated approach, focusing on earlier interventions, biomarker-driven precision treatment, and improved drug delivery systems, such as subcutaneous injections, receptor-mediated transport, and antibodies with enhanced BBB penetration. As it stands, high treatment costs, limited accessibility, and strict eligibility criteria all stand as barriers to treatment. By integrating the *APOE4* genotype into treatment planning and focusing on disease-stage-specific approaches, a safer and more effective means of treating AD could be achieved.

## 1. Introduction

According to the National Institute of Aging, Alzheimer’s disease (AD) is the “seventh leading cause of death in the United States,” presenting mostly in the aging population [1]. Though its pathogenesis is not well understood, diffuse plaques of extracellular amyloid-beta peptide (Aβ) deposition and neurofibrillary tangles, aggregates of tau protein, are hallmark neuropathologic changes found in AD [2,3]. All AD cases have either an overproduction or a decreased clearance of amyloid peptides; hence, antibodies against these have been developed, with various degrees of success. It has been highlighted that small aggregates rather than large aggregates (fibrils) are the main neurotoxic culprit in AD. A risk gene, identified as *APOE4*, which produces apolipoprotein E4 (apoE4) in the central nervous system (CNS), impairs Aβ clearance from the cerebrum; hence, being a carrier may impair response to treatments.

The importance of apoE was first established in peripheral lipid transport, in the context of hyperlipidemia, with the discovery that cholesterol-rich particles attached to arginine-rich proteins (apoE) were present in elevated levels in the plasma [4]. Normally, apoE plays important roles in neural and vascular health, with primary functions in cholesterol transport, lipid metabolism, and the clearance of Aβ [5]. In the brain, apoE is produced primarily by astrocytes, the choroid plexus, microglia, and neurons responding to injury or growth [6]. In the CNS, apoE helps to transport lipids to repair membranes and build myelin sheaths.

Two of *APOE*’s isoforms, *APOE2* and *APOE3*, are common and even provide some protection against the progression of AD and other neurodegenerative disorders. *APOE3*, for example, helps prevent synaptic loss by increasing the expression of protein kinase Cε, which promotes the cleavage of amyloid precursor protein via α-secretase, in turn reducing the production of Aβ peptides and plaques [7,8]. Furthermore, *APOE3* has been found to enhance nerve sprouting, play a role in axonal growth and regeneration, and its protein was found in neutrophils and macrophages during neural remodeling following spinal cord injury [9]. However, when investigating AD, the primary gene of interest tends to be *APOE4*, as its presence correlates with hallmark symptoms of the disease [10,11].

While *APOE2* and *APOE3* tend to be beneficial, the ε4 isoform of *APOE* has been identified as the primary genetic risk factor for AD [10]. In some populations, 40–80% of affected individuals possess at least one *APOE4* allele [12]. In investigating its functional link to AD, *APOE4* has been associated with, though not limited to, the following: impaired utilization of glucose in the CNS, blood–brain barrier (BBB) breakdown, increased levels of intracellular Aβ, and the phosphorylation of tau proteins [5,12,13].

There are several ways in which *APOE4* is thought to be involved with increased levels of Aβ, namely that it enhances the expression of amyloid-beta-precursor protein (APP), lowers the expression of protective enzymes, affects the formation of Aβ oligomers, and decreases Aβ clearance through the BBB. Firstly, by enhancing the expression of APP and thus increasing the accumulation of Aβ in the brain’s interstitial fluid, *APOE4* effectively exceeds the intracellular lysosomal limit of Aβ degradation, leading to neuronal toxicity [14]. Secondly, *APOE4* lowers the expression of the enzyme neprilysin, which normally degrades Aβ [5,15,16]. In mouse models, the inhibition of neprilysin function increased in total brain Aβ levels, specifically in hippocampal CA1 neurons [17]. Thirdly, in binding to Aβ oligomers, nuclei form, which are required for fibril growth. apoE4 can thus accelerate the formation and growth of fibrils, and can later stabilize them, which makes them difficult to break down and more likely to form plaques [18]. Lastly, since the binding of Aβ to apoE4 favors internalization via very-low-density lipoprotein receptors (VLDLRs) instead of LRP1-mediated internalization, the BBB clearance of Aβ is disrupted, so Aβ and Aβ-apoE4 complexes build up in the interstitial fluid [5,19].

Further research has also demonstrated that *APOE4* may play a role in tau pathology, which, as mentioned previously, is another pathological hallmark of AD. C-terminal truncated apoE4 fragments from neuronal cleavage can stimulate the phosphorylation of tau proteins, which can form neurofibrillary tangle precursors, in turn causing cytoskeleton depolymerization and the inhibition of axonal growth [20].

Now that the relationship between *APOE4* and Aβ accumulation has been more clearly established, it would be prudent to examine the links between these mechanisms and vascular abnormalities, specifically amyloid-related imaging abnormalities (ARIA). To begin, both preclinical and clinical trials have demonstrated that *APOE4* is a major risk factor for anti-Aβ immunotherapy-induced ARIA, and this can be detected by Magnetic Resonance Imaging (MRI) and classified in two forms: ARIA-E (edema) and ARIA-H (hemorrhage) [21].

The strongest connection between the mechanisms above and ARIA is seen with impaired BBB clearance of Aβ. By increasing the overall levels of Aβ and diminishing BBB clearance thereof, *APOE4* can lead to and is the strongest genetic risk factor of cerebral amyloid angiopathy (CAA)—too much Aβ is circulating in the interstitial fluid, and it then builds up along vessel walls [22]. The vascular dysfunction caused by CAA reduces perivascular Aβ clearance, which leads to even more buildup. Anti-Aβ immunotherapy then mobilizes the built-up parenchymal Aβ. The often-observed ARIA could be a result of the mobilization of these plaques into the perivascular drainage system, which also starts an immune response. All of this together overwhelms the vessels’ Aβ clearance capacity, leading to deposition of Aβ and plaques in the arterial walls and worsening of CAA. This disrupts vascular integrity, which can lead to leakage of proteinaceous fluid (ARIA-E) and red blood cells (ARIA-H).

There are several possible mechanisms leading to apoE4 being a noteworthy risk factor for AD, as well as a risk for developing ARIA when on anti-amyloid therapies. The first of these, decreased astrocyte-blood vessel contacts due to abnormal apoE4 altering lipid metabolism, triggers increased BBB leakiness, resulting in the observed ARIAs. Secondly, apoE4 carriers are more likely to have increased amyloid oligomer burden, which results in increased blood vessel integrity disruption when exposed to anti-amyloids. Thirdly, “*APOE4* increases the likelihood of CAA, suggesting that the *APOE4* effect may be mediated by the increased presence of CAA among *APOE4*-positive individuals, even in the absence of radiological evidence” [23].

Several acquired AD risk factors include hypertension, dyslipidemia, cerebrovascular disease, type 2 diabetes, obesity, lifestyle, and medications (like anticholinergics and PPIs). Diagnosis of AD is made when the patient experiences a decline in both cognition and function, along with imaging-based evidence of specific neuropathology [2].

There are several treatments given to patients with AD, including cholinesterase inhibitors, memantine, antioxidants, amyloid-targeted therapies, and medications for symptom and vascular risk management [23]. The cholinesterase inhibitors are administered due to reduced choline acetyltransferase activity in the cerebrum of AD patients, which decreases acetylcholine synthesis and, hence, impairs cholinergic function. Memantine may also be administered as an N-methyl-D-aspartate (NMDA) antagonist, which may protect the brain from excessive NMDA receptor stimulation, thereby potentially mitigating ischemia and excitotoxicity. Amyloid-targeted therapies aim to reduce amyloid plaque burden and possibly be disease-modifying rather than being purely symptomatic treatment [24].

The drugs reviewed in this article are aducanumab, lecanemab, and donanemab—all monoclonal antibodies produced within the last decade, acting as passive immunization against amyloid-peptides and were approved by the Food and Drug Administration (FDA) a federal agency of the United States Department of Health and Human Services. Aducanumab is a fully human anti-Aβ IgG1, administered intravenously (IV) every 4 weeks, typically at a dose of 10 mg/kg. It binds to aggregated forms of Aβ, reducing the number of plaques and potentially slowing disease progression. It has a “higher affinity for fibrillar aggregates compared with monomers” [25]. It received accelerated approval in June 2021 by the FDA, becoming the first approved Aβ-targeting monoclonal antibody approved for AD; it was later also approved in the UAE. Lecanemab, a humanized IgG1 monoclonal antibody, binds with high affinity to Aβ soluble protofibrils, administered IV without titration at a dose of 10 mg/kg biweekly. It was granted FDA accelerated approval in January 2023 and full FDA approval in July 2023 for patients with Aβ-positive mild AD dementia or MCI (mild cognitive impairment); it is yet to be approved in the EU. Donanemab (LY3002813; N3pG), given as a once-monthly injection, is another humanized IgG1 monoclonal antibody derived from mouse mE8-IgG2a, which binds to Aβ plaque, specifically AβpE, and initiates its clearance by microglia through recognizing the N-terminal pyroglutamate Aβ [26]. It is FDA-approved as of 2 July 2024.

The objective of this narrative review is to examine the development and possible role of these FDA-approved and future anti-Aβ monoclonal antibodies in the care of AD patients, considering the current state of knowledge and limitations. We conducted a comprehensive literature search of PubMed, Web of Science and Scopus databases related to the development of aducanumab, lecanemab, and donanemab. The timeframe of clinical trials discussed spans as early as 2009, with the majority of trials beginning in 2022 and 2023. The supporting literature cited dates back to 2002, with the majority of studies falling within the 2012–2025 range.

## 2. From Clinical Trials to FDA Approval

### 2.1. Aducanumab

#### 2.1.1. Phase I Clinical Trials

**Figure 1:**
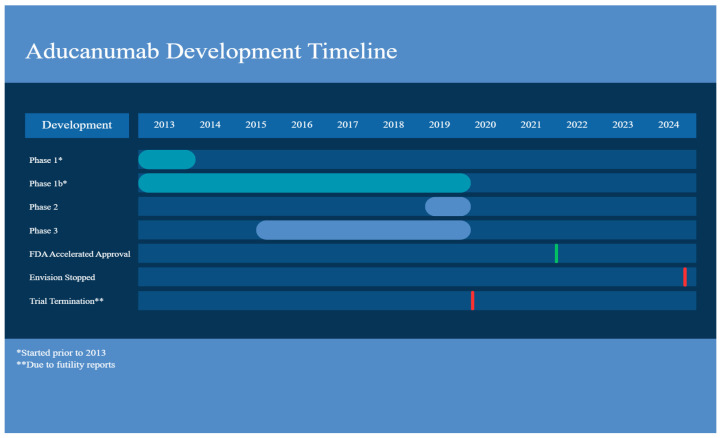
Aducanumab development timeline. Created with Canva.

Phase I (NCT01397539):

This clinical trial evaluated the safety and tolerability of a single intravenous (IV) infusion in participants with AD. In this double-blind placebo-controlled trial (human) with aducanumab doses of 0.3, 1, 3, 10, 20, 30 mg/kg being given, participants were between 55 and 85 years old and required to have a MMSE (Mini-Mental State Examination) score between 14 and 26 (namely mild/moderate AD). The dose of aducanumab ≤ 30 mg/kg was well tolerated with no ARIA and SAEs; while the dose of 60 mg/kg resulted in ARIA, which resolved by weeks 8–15 [27]. Many common adverse events (AEs) were reported and these included headaches, upper respiratory tract infections, and diarrhea. The increase in dose did not change overall clearance, suggesting linear pharmacokinetics [27] (Figure 1) (Table 1).

Phase Ib (NCT01677572):

PRIME was designed to investigate the safety, tolerability, and pharmacokinetics of aducanumab injections in those with mild AD with Aβ as observed on Positron Emission Tomography (PET) imaging. In this placebo-controlled trial, doses of 1, 3, 6, and 10 were administered IV every 4 weeks for 1 year. The decrease in brain Aβ biomarker observed on PET was dose—and time-dependent; decreases in clinical worsening, as measured by CDR-SB and MMSE, were also observed. The antibody was found not to bind to soluble Aβ monomers, hence plasma concentrations remained stable. In the 10 mg/kg group, 47% of the participants developed ARIA; however, it resolved within 4–12 weeks [29]. 

#### 2.1.2. Phase II Clinical Trials

Phase II (NCT03639987) EVOLVE:

The main objective of this trial was to determine the safety of continuing aducanumab dosing in participants with MCI due to AD experiencing asymptomatic ARIA. Two groups (*n* = 26 each) had the aducanumab dose titrated up to 10 mg/kg via IV infusion and were followed up to week 54 with different ARIA management strategies [25]. The trial was stopped in July 2019 based on futility analysis performed on Phase III trials. Before its termination, 7.7% of Group 1 was found to have ARIA on MRI (NCT03639987).

#### 2.1.3. Phase III Clinical Trials

Phase III ENGAGE (NCT02477800) and EMERGE (NCT02484547):

Two randomized, double-blind, placebo-controlled global Phase III trials were conducted with early AD patients, EMERGE, with 1638 participants, and ENGAGE, with 1638 participants. The trial’s inclusion criteria were MMSE 24–30 (mild AD), clinical dementia rating sum of boxes (CDR-SB) 0.5, and a positive PET. The primary objective was to determine if aducanumab reduced cognitive impairment, measured in CDR-SB, which stages the severity of AD and MCI, compared to placebo. The participants were split into 3 groups: low dose (3 mg/kg for *APOE4* carriers, 6 mg/kg for non-carriers), high dose (6 mg/kg for *APOE4* carriers, 10 mg/kg for non-carriers), and placebo. Aducanumab was administered every 4 weeks for 76 weeks. The two trials had different degrees of success, with both observing a dose and time-dependent reduction in pathophysiological markers of AD. In EMERGE, the high-dose aducanumab arm was observed to have a 22% reduction in decline in CDK-SB from baseline (the primary endpoint) at week 78, along with decreases in secondary outcomes like MMSE [30]. ENGAGE met none of its setpoints, however.

Aducanumab successfully reduced plaques along with CSF tau and tau PET biomarkers, both of which decreased in a dose-related manner. The CSF p-tau levels were observed in both studies to be reduced by 22.44 pg/mL in EMERGE and 13.19 pg/mL in ENGAGE when the high dose of the mAb was used. The plasma p-tau biomarker decreased by 13% and 16% from baseline in the EMERGE and ENGAGE studies, respectively [25].

The most common adverse effect, ARIA-E, affected 35.2% of patients on the highest dose of aducanumab (10 mg/kg), with 263 events occurring within the first 8 infusions with aducanumab. About a quarter of those patients were symptomatic. The most common associated symptoms in these patients included headache (46.6%), confusion (14.6%), dizziness (10.7%), and nausea (7.8%) [31]. Additionally, ARIA-microhemorrhages affected 197 of 1029 patients in the 10 mg/kg group (19.1%) and 71 of 1076 participants taking the placebo (6.6%); ARIA-superficial siderosis occurred in 151 participants in the 10 mg/kg group (14.7%) and 24 patients in the placebo group (2.2%) [31]. In the group administered 10 mg/kg, 43% of *APOE4* carriers developed ARIA-E, and only 20.3% of non-carriers did. In the placebo group, 2.7% developed ARIA-E, with specifically 2.2% of carriers and 3.9% of non-carriers being affected. Ninety-eight percent of ARIA-E resolved radiographically, with 82.8% clearing up within 16 weeks [31].

A Markov modeling approach used to predict the long-term benefits of aducanumab using EMERGE efficacy data found that the treatment was associated with “0.65 incremental patient quality-adjusted life-years (QALYs) and 0.09 fewer caregiver QALYs lost compared with patients treated with standard of care” [28]. Additionally, the treatment was determined to lower the lifetime probability of “transitioning to AD dementia, a lower lifetime probability of transitioning to institutionalization (25.2% vs. 29.4%), and an incremental median time in the community of 1.32 years compared with” patients receiving standard care alone [28].

Nevertheless, in March 2019, ENGAGE and EMERGE were terminated due to futility analysis rather than safety concerns (Figure 1).

Aducanumab Appropriate Use Recommendations (AURs) involve clinician watchfulness of ARIA, which includes performing APOE genotyping to ensure better patient care and awareness of risk; it is also recommended to perform MRIs before the 5th, 7th, 9th, and 12th infusions to improve ARIA detection due to most ARIAs occurring during the titration period [32].

While both EMERGE and ENGAGE trials were halted due to futility reports, EMERGE suggested a modest benefit at a high dose, a 22% relative reduction in clinical dementia score; ENGAGE showed less clinical benefit, making the accelerated approval questionable. Biogen relied on post hoc and subgroup analyses to justify approval; however, post hoc analyses should be used to create hypotheses rather than to decide drug benefit. Some argue that FDA’s approval is even unethical as the magnitude of the benefit outweighing the risk of ARIA is poorly understood. Additionally cultivating false hope and its high costs on American healthcare adds to the unethical claim [33]. Many other factors add to the hesitation around the approval, including the drug being approved for all patients with AD rather than the subgroup studied in the clinical trial, namely, early Alzheimer’s. Others contend that the efficacy of the surrogate biomarker, amyloid plaques, is questionable. The FDA’s own Peripheral and Central Nervous System Advisory Committee unanimously agreed that the though results show possible clinical effect, it did not show proof of effect on disease progression. The positive result in EMERGE “when considered with the totality of the evidence, did not amount to the substantial evidence of efficacy from adequate, well-controlled trials that the law requires and that patients and physicians should expect for traditional approval” [34]. Additionally, the committee was not consulted regarding the appropriateness of Aβ as a surrogate biomarker, with an effective one expected to have a strong correlation with the clinical endpoint. The number of amyloid plaques does not correlate well with disease progression, rendering it a poor surrogate marker. Hence, FDA’s accelerated approval was surprising and controversial, with some criticizing FDA’s questionable close interaction with Biogen during the review process. While accelerated approval requires confirmatory studies, such studies practically take many years to complete and are often delayed or fail [35]. Aaron Kesselheim, who had resigned from the FDA Peripheral and Central Nervous System Advisory Committee after aducanumab was approved, publicly stated that this was ‘probably the worst drug approval decision in recent U.S. history. David Knopman and Joel Perlmutter also resigned from the committee, emphasizing a lack of strong efficacy evidence and criticizing the FDA for overriding the panel’s recommendation against approval.

On 31 January 2024, the ENVISION trial, a requirement for aducanumab accelerated approval, was announced to be stopped by Biogen in May 2024, with rights going back to Neurimmune, which plans to create a formulation of the drug for subcutaneous administration. It should be noted that the FDA has not, as of the writing of this article, canceled its accelerated approval of the drug [36] (Table 1).

### 2.2. Lecanemab

#### 2.2.1. Phase I Clinical Trials

**Figure 2:**
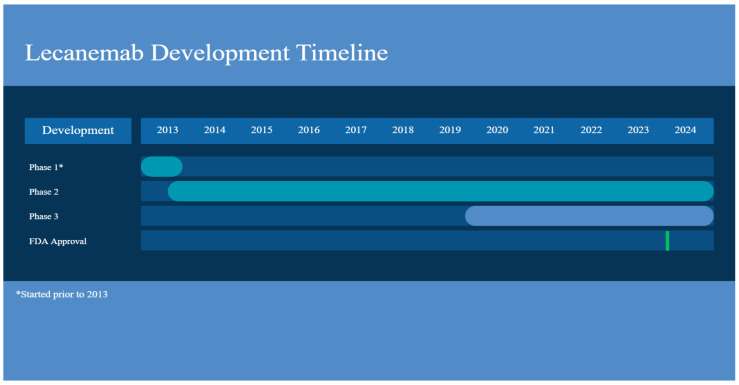
Lecanemab development timeline. Created with Canva.

Phase I (NCT01230853):

The first clinical study with lecanemab (BAN2401), which enrolled 80 participants to determine its safety and tolerability at ascending doses in AD patients, was randomized, double-blind, and placebo-controlled. This clinical trial consisted of two parts: single ascending dose (SAD) and multiple ascending dose (MAD). The SAD study, investigated doses of 0.1, 0.3, 1, 3, 10, and 15 mg/kg, while MAD, after doses were observed to be well-tolerated in the SAD cohort, evaluated infusions of “0.3, 1, and 3 mg/kg administered every four weeks with a total of four doses over four months and a dose of 10 mg/kg biweekly, with a total of seven doses over four months” [37] (Figure 2) (Table 1).

No trends in the SAD cohort were found to suggest increased treatment-emergent adverse events (TEAEs) with increased dose; dizziness, fatigue, and sinusitis were found to be the most common TEAEs, with only sinusitis occurring in a higher percent in those receiving the dose compared to placebo, 5.6% and 0%, respectively. “Upper respiratory tract infection (16.7% vs. 12.5% on placebo), headache (12.5% vs. 25% on placebo), and orthostatic hypotension (12.5% vs. 0% on placebo)” were common in those receiving multiple doses of BAN2401 [37]. For the SAD and MAD cohorts, all TEAEs were considered mild or moderate. Asymptomatic ARIA-H was observed in 5% (3/60) receiving the treatment, as opposed to the 10% (2/20) observed in the placebo group. 

The drug exposure over time, the area under the curve (AUC), was “approximately dose proportional”; the serum half-life after the final dose was found to range from 5.3 in the 10 mg/kg biweekly MAD cohort to 7.3 days “in the 15 mg/kg SAD cohort, with first-order kinetics elimination [37]. Steady state concentrations were reached after the third dose of 10 mg/kg administered biweekly. Additionally, BAN2401 was found to enter the BBB and be measurable in the CSF. In summary, BAN2401 was found to be well-tolerated at all doses tested, and the maximum tolerated dose was not reached in either SAD or MAD.

#### 2.2.2. Phase II Clinical Trials

Phase II (NCT01767311):

The Phase IIb, an 18-month trial, was performed to determine the dose and efficacy of the lecanemab treatment. The study enrolled 856 participants who were required to have “Aβ pathology confirmed by PET or CSF Aβ_1–42_ measurement, an MMSE ≥  22 (22–28 in participating EU nations), and objective memory impairment (Weschler Memory Scale IV–Logical Memory II [WMS-IV LMII]) criteria” [25]. Of the 854 treated, 609 were given lecanemab and 245 a placebo.

The trial was unsuccessful at meeting its primary endpoint at 12 months with the dosage of 10 mg/kg biweekly, resulting in a 64% rather than the required 80% “probability of slowing Alzheimer’s disease composite score (ADCOMS) decline by 25% more than placebo” [25]. Nevertheless, additional prespecified Bayesian analyses found this dosage of lecanumab had a 97.6% and 97.7% probability of being more effective than placebo, at 12 months and 18 months, respectively. At 18 months, the lecanemab 10 mg/kg biweekly dose, according to a prespecified Bayesian analysis, was shown to have a “76% probability of being better than placebo” at slowing ADCOMS decline by 25%. “10-mg/kg biweekly lecanemab reduced brain amyloid (−0.306 SUVr units) while showing a drug-placebo difference in favor of active treatment by 27% and 30% on ADCOMS, 56% and 47% on ADAS-Cog14, and 33% and 26% on CDR-SB versus placebo according to Bayesian and frequentist analyses, respectively” [38]. With regard to dosage, placebo and the 3 lowest doses, 2.5 mg/kg biweekly, and 5 mg/kg biweekly and monthly, did not differ. On CDR-SB in 18 months, clinical decline was reduced by 26% at 10 mg/kg biweekly and 17% with 10 mg/kg monthly, compared to placebo, making 10 mg/kg biweekly the optimal dose [38]. In addition, regarding the biomarkers, 10 mg/kg dose arms resulted in “an increase in CSF Aβ42 and decrease in p-tau relative to placebo, whereas inconsistent results were noted at 12 months and 18 months for total tau” [38].

The common adverse effects of lecanemab treatment were infusion reaction (3.3% and19.9% for placebo and 10 mg/kg biweekly, respectively) and ARIA-E (0.8% and 9.9% of participants for placebo and 10 mg/kg biweekly, respectively). An amendment to the trial required the removal of *APOE4* carriers from the 10 mg/kg biweekly group due to increased risk of ARIA.

There are currently Phase II/III multicenter randomized, double-blind, placebo-controlled trials, one active and the other recruiting (NCT05269394 and NCT01760005, respectively), with the aim of studying therapies in individuals with the DIAN-TU mutation, using biomarker, cognitive, and clinical endpoints [39].

#### 2.2.3. Phase III Clinical Trials

Phase III Clarity AD (NCT03887455):

The Clarity AD, a placebo-controlled, double-blind, parallel group study on early AD patients, evaluated the efficacy of lecanemab compared to placebo using the change from baseline in CDR-SB at 18 months. The participants were randomized 1:1 into two groups to be administered IV10 mg/kg biweekly lecanemab (*n* = 898) or placebo (*n* = 897). The following were some criteria to be eligible for this trial: “age (50–90 years), an MCI or mild AD diagnosis (National Institute on Aging–Alzheimer’s Association [NIA-AA] criteria), a 1 standard deviation (SD) decrease in objective episodic memory below the age-adjusted mean (WMS-IV LMII), and Aβ biomarker positivity by PET or CSF Aβ_1–42_ measurement” [25].

Both groups had mean CDR-SB scores of 3.2 at baseline. The mean change in CDR-SB from baseline was 0.45 lower in those who received lecanemab (1.21) as opposed to placebo (1.66). A substudy of 698 found brain amyloid biomarker levels to be reduced to a greater degree in those in the lecanemab arm. The statistically significant mean differences in change between the two groups from baseline found in the secondary endpoints also support the efficacy of lecanamab: “the ADAS-cog14 score, −1.44; for the ADCOMS, −0.050; and for the ADCS-MCI-ADL score, 2.0” [40].

With all its benefits for AD patients, lecanemab also has adverse effects, including “infusion-related reactions in 26.4% of the participants and amyloid-related imaging abnormalities with edema or effusions in 12.6%” [40]. Incidences of ARIA in the lecanemab arm were found to be higher in carriers of the *APOE4* allele in the lecanemab arm as opposed to non-carriers, ARIA-H: 14%, ARIA-E: 10.9% and ARIA-H: 11.9%, ARIA-E: 5.4%, respectively. Homozygotes for the allele had an even higher incidence of ARIA-H and ARIA-E, 39% and 32.6%, respectively [25].

Based on the results of this phase, lecanemab was granted standard approval by FDA, following accelerated approval based on the Phase IIb trial (Figure 2).

Phase III AHEAD 3–45 (NCT04468659):

Another Phase III trial currently recruiting (NCT04468659) is the AHEAD 3–45 study, “a placebo-controlled, double-blind, parallel-treatment arm, 216-week study with an extension phase to evaluate efficacy and safety of treatment with BAN2401 in subjects with preclinical Alzheimer’s disease and elevated amyloid (A45 Trial) and in subjects with early preclinical Alzheimer’s disease and intermediate amyloid (A3 Trial)” [41]. This study will test whether lecanemab can prevent cognitive decline along with tau accumulation (Table 1).

### 2.3. Donanemab

#### 2.3.1. Phase I Clinical Trials

**Figure 3:**
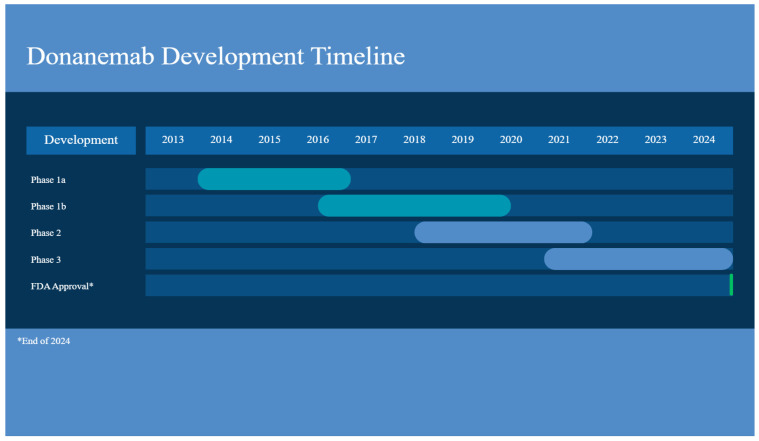
Donanemab development timeline. Created with Canva.

Phase Ia—NCT01837641:

This study, aimed to explore the donanemab’s safety and tolerability, was “a subject- and investigator-blind, randomized, placebo-controlled, parallel-group, single-dose followed by a multiple-dose, dose-escalation study” [42]. The participants with MCI due to AD were put into a SAD phase (with five cohorts from 0.1 to 10 mg/kg and one placebo), being administered a single IV dose, preceding a 12-week follow-up; they were then put into a MAD phase, being given a dose once a month. The antibody was well tolerated by humans up to 10 mg/kg. The mean terminal elimination half-life after administration from 0.1 to 3.0 mg/kg was about 4 days, with it being 10 days at 10 mg/kg. Ten mg/kg IV monthly was the only dose to show changes in amyloid PET, with a 40–50% reduction in Aβ plaques [42]. Unfortunately, at 3 months after a single IV dose, 90% of patients developed anti-drug antibodies. Additionally, ARIA-H was experienced by 2 patients; however, there were no reports of ARIA-E [42] (Figure 3) (Table 1).

Phase Ib—NCT02624778:

The phase Ib trial explored the effect of the drug on amyloid plaque levels, in an “investigator- and patient-blind randomized, placebo-controlled study” [43]. SAD and MAD studies enrolled 61 participants with MCI due to AD (and are amyloid plaque positive) in 6 cohorts “with doses ranging from 10 to 40 mg/kg administered through IV infusion as a single or multiple dose regimen. A reduction in plaque was observed in the multiple-dose cohorts by 24 weeks. Dose-proportional increases in pharmacokinetics were found from 10 to 40 mg/kg for single doses and at steady state for multiple-dose cohorts. As with Phase Ia, more than 90% of patients developed antidrug antibodies. Donanemab, while well tolerated, was observed to result in “12 vasogenic cerebral edema events (12 [19.7%] patients) and 10 cerebral microhemorrhage events (6 [13.0%] patients)”; ARIA- E was reported in 26% of those receiving donanemab [43].

#### 2.3.2. Phase II Clinical Trials

Phase II TRAILBLAZER-ALZ (NCT03367403):

The 272 early symptomatic AD patients in the TRAILBLAZER-ALZ study involved 131 participants receiving donanemab 700 mg IV every 4 weeks for 3 doses, then 1400 mg IV every 4 weeks until week 72, and 126 receiving placebo IV every 4 weeks for up to 72 weeks, and 15 receiving donanemab in combination with LY3202626 (donanemab-C) [44]. The randomized, double-blind, Phase II clinical trial inclusion criteria included an MMSE score of 20–28 and Aβ pathology by PET, with no history of long QT syndrome.

After treatment with donanemab, the Integrated Alzheimer’s Disease Rating Scale (iADRS) score was shown to slow down by 25%. The change from baseline in the iADRS score at 76 weeks was −6.86 with donanemab and −10.06 with placebo (difference, 3.20; 95% confidence interval, 0.12 to 6.27; *p* = 0.04). At 76 weeks, when compared to placebo, amyloid plaque levels reduced by 85.06 centiloid, “with 67.8% of participants having negative Aβ status [25].

#### 2.3.3. Phase III Clinical Trials

Phase III TRAILBLAZER-ALZ 2 (NCT04437511):

TRAILBLAZER-ALZ 2 trial enrolled 1800 participants with inclusion criteria similar to the Phase II trial previously described, but broadened to include individuals with high levels of tau to evaluate the effects of donanemab in this population.

Participants identified to have intermediate tau biomarker levels and clinical symptoms of AD and who were on donanemab had a slowing of decline of 35% in iARDS and 36% in CDR-SB over 18 months [45]. Those on the drug compared to placebo showed a 39% lower risk of progressing to the next stage of disease [45]. ARIA-E and -H occurred in 24% and 31.4% of participants treated with donanemab, “with 6.1% experiencing symptomatic ARIA-E” [45]. Homozygous and heterozygous *APOE4* carriers experienced ARIA-E at a higher frequency than noncarriers, 40.6% and 22.8% as opposed to only 15.7% [46]. After the FDA rejection of donanemab for accelerated approval due to insufficient safety data in Phase II, Eli Lilly applied for traditional approval due to the Phase III TRAILBLAZER-ALZ 2 clinical trial’s positive outcomes [25]. Donanemab (brand name Kisunla), received traditional approval from the FDA on 2 July 2024 (Figure 3).

Phase III TRAILBLAZER-ALZ 3 (NCT05026866):

An incremental Phase III study TRAILBLAZER-ALZ 3 is active, but no longer recruiting, with 2196 preclinical AD participants with the presence of Aβ and early-tau pathology. Its focus is to evaluate the safety and efficacy of donanemab in this demographic, with the primary outcome being measured in Clinical Dementia Rating-Global Score (CDR-GS).

Phase III TRAILBLAZER-ALZ 4 (NCT05108922):

Another Phase III, open-label, two-arm comparison study recruited 200 participants with early symptomatic AD and abnormal biomarker Aβ levels on amyloid PET to compare the effects of donanemab with aducanumab. Of the participants in the study, 37.9% treated with donanemab achieved clearance, while only 1.6% of those taking aducanumab did [47]. Additionally, the percentage of those who had reported adverse effects was higher for those taking aducanumab compared to donanemab, 66.7% and 62%, respectively; there were no serious adverse effects in donanemab and only one event in those taking aducanumab.

Phase III TRAILBLAZER-ALZ 6 (NCT05738486):

TRAILBLAZER-ALZ 6 is an active not recruiting trial, has 800 AD adults with amyloid pathology enrolled to investigate multiple dosing regimens and their effect on ARIA-E, both in terms of frequency and severity, to determine predictors of ARIA risk [48].

Phase III TRAILBLAZER-ALZ 5 (NCT05508789):

TRAILBLAZER-ALZ 5 is currently recruiting 1500 participants with early AD. This is a double-blind, placebo-controlled, Phase III trial aiming to assess the safety and efficacy of donanemab, using the iADRS score as a primary outcome measure, and CDR-SB as one of the secondary outcome measures [49] (Table 1).

## 3. Discussion and Conclusions

### 3.1. mAb Treatments and APOE4 Carriers

The three FDA-approved drugs, aducanumab, lecanemab and donanemab showed a decent reduction in the decrease in CDR-SB, while ARIA-E remains a major concern for all three, especially in the *APOE4* carriers (Table 1). Many trials with these drugs are still in progress, some are still recruiting, to understand the efficacy and safety of the drugs in AD patients. Additionally, there are currently many new drugs under trial, some following similar mechanisms to the above-mentioned, such as remternetug, and others targeting other pathways, like blarcamersine, which activates the sigma-1 receptor to prevent amyloid misfolding. Further research is needed to compare treatments and explore dual treatments, determining which drug dosing and combinations provide the best outcome with minimal adverse effects for all.

For all three mAb therapeutics, aducanumab, lecanemab and donanemab, the adverse effects of concern for *APOE4* carriers are ARIA. In those receiving aducanumab, the percentage of carriers with ARIA-E compared to non-carriers was doubled; a similar trend was found in lecanumab. Incidences for aducanumab were dose dependent, with 55% of *APOE4* carriers in the high-dose aducanumab group being affected; withholding treatment and sometimes administering corticosteroids was found to resolve most ARIA-E cases [50]. When treated with lecanumab, ARIA-E was found to correlate with serum concentration; this adverse effect was also higher in incidence for homozygous *APOE4* carriers versus heterozygous carriers and non-carriers [51]. The same pattern is observed when studying donanemab: homozygous and heterozygous *APOE4* carriers experience ARIA-E at a higher frequency than non-carriers, 40.6% and 22.8%, respectively, compared to 15.7%. A Phase III trial with another humanized anti–Aβ monoclonal antibody, bapineuzumab, not currently FDA approved, highlighted an overall higher incidence proportion of ARIA-E in *APOE4* carriers as opposed to non-carriers; additionally, it was higher in homozygotes than in heterozygotes (34.5% versus 16.9%) [52]. On the other hand, acetylcholinesterase inhibitors, in a meta-analysis by Cheng et al. were found to have a positive therapeutic effect compared with placebo regardless of *APOE4* status of AD patients [53]. Across anti-amyloid monoclonal antibodies, *APOE4* status consistently correlates with risk for ARIA. For aducanumab and donanemab, *APOE4* carriers—particularly homozygotes—exhibit substantially higher ARIA-E and ARIA-H incidence compared to non-carriers, whereas lecanemab shows a lower overall ARIA burden. It does, however, retain clear genotype-dependent risk. Collectively, these findings position the *APOE4* genotype as a key determinant of anti-amyloid therapy safety, reinforcing the need for genotype-informed patient selection, monitoring and treatment.

Going past informing of ARIA risk, *APOE4* genotyping could be integrated into clinical decision-making to guide patient selection and the type and timing of treatment options, since *APOE4* carriers—homozygotes especially—have distinct disease trajectories, biomarker profiles, and treatment responses. Incorporating *APOE* status along with amyloid or tau biomarkers may enable earlier intervention, risk–benefit counseling, and prioritization of genotype-tailored or non-amyloid-based therapies for high-risk individuals.

Interestingly, a systematic review found exercise to be a non-pharmacological treatment option for high-risk *APOE4* carriers, as it influences AD pathology, including reducing Aβ load, protecting against hippocampal atrophy, improving cognitive function, stabilizing cholesterol levels and lowering pro-inflammatory signals [54]. A study on apoE4-targeted replacement mice found that when the anti-apoE4 antibody, mAb 9D11, is applied intracerebroventricularly, it prevents the apoE4-driven accumulation of Aβ in hippocampal neurons. When injected peripherally, it was associated with reversal of cognitive impairments [55].

These findings suggest patient *APOE4* status should be considered in the management of AD to ensure safe, optimized treatment. Additionally, further research into anti-apoE4 immunotherapies, as well as promotion of a healthy lifestyle, would aid *APOE4* carriers in their disease management, possibly reducing risk and improving symptoms.

### 3.2. Overview and Moving Forward

In the last three to five years, especially, AD research has entered a more translational phase, marked by the regulatory approval of mAbs like aducanumab, lecanemab, and donanemab, alongside a growing recognition of genotype-dependent treatments and treatment effects. Similarly, clinical cognitive and biomarker data have more clearly established that *APOE4* homozygosity is a major contributor to and modifier of disease trajectory, ARIA risk, and therapeutic responses. This prompts the need for more closely monitored regulatory guidance and trial designs that explicitly incorporate *APOE* genotyping.

Although several anti-amyloid drugs have been developed for treating and slowing AD progression, only a few, such as aducanumab, lecanemab, and donanemab, have been approved and have shown benefits beyond modest cognitive and functional improvements. Each of these drugs has been shown to treat early and mild cases of AD, acting through the targeting of Aβ aggregates, Aβ protofibrils, and pyroglutamate-modified Aβ, respectively [56]. The vast majority of drugs being developed to treat AD are anywhere from Phase I to III of approval, and the treatment targets vary greatly—from Aꞵ aggregates and p-tau to insulin receptors or plasma factors (e.g., CM383, JNJ-63733656, insulin, and ExPlas, respectively), just to name a few [56]. This demonstrates that a diverse array of treatments is being investigated, as the causes, neurological symptoms, and cellular pathologies of AD are many that are still fully being explored.

Most drugs progressed to Phase II or III, targeting various forms of tau, fibrillar Aβ, soluble Aβ, Aβ oligomers, and aggregated Aβ, among others. Outlined reasons for discontinuation primarily center around a lack of observed cognitive benefits, with endpoints not being met and biomarker benefits lacking. Some drugs targeting Aꞵ, like gantenerumab, were successful in reducing plaque; however, there was little positive change from participants’ baseline PACC-5 score, and in some cases, they showed further cognitive decline [57]. Others, such as ponezumab, which targeted soluble Aꞵ, showed no biomarker or cognitive benefits to treatment, with baseline amyloid load remaining largely unchanged [58].

In moving forward with future research, given the success of some treatments and the failures and risks of others, several shifts are occurring in the field. The therapeutic targets are being reevaluated, and new targets are being examined, which include neuroinflammation, vascular dysfunction and BBB breakdown, endocrine dysregulation (including insulin resistance), and lipid metabolism (with a focus on apoE). In exploring such avenues and focusing on new targets, the different underlying pathologies of AD need to be better understood and treated, which could lead to a better systematic treatment of the disease, rather than merely focusing on the better-established targets of Aβ and tau. The primary barrier that remains to be overcome is that many drugs, especially those that investigate the aforementioned targets, cannot be readily delivered to the brain. The BBB is not easily crossed, and even with intravenous drugs like lecanemab and donanemab, only a small portion actually reaches the brain and target tissues therein. This results in higher risks of peripheral side effects, increased healthcare costs, and a significant treatment burden.

Additionally, computational drug discovery—which provides a method for determining, in silico, which molecules are most likely to interact with therapeutic targets—offers a promising route to discovering treatments for new therapeutic targets of AD [59]. Though this method has been utilized for targets like Aβ, tau, and apoE, it is being further applied to examine novel targets like acetylcholinesterase, β-secretase, monoamine oxidase-B, and many more [59,60,61]. With molecular modeling, ligand-based approaches, simulations of treatment, and other computer-aided forms of drug design, computational drug discovery provides a means to reduce both the time and costs that go into searching for effective treatments for AD [61].

Even for approved drug treatments, there are still numerous barriers to receiving treatment. Broadly stated, access to adequate treatment facilities, early and accurate diagnosis of AD, and updated clinical guidelines are necessary for these treatments to be effective [62]. For example, following the injection of lecanemab or donanemab, patients must undergo MRI monitoring to detect ARIA. In countries like Japan, which leads the world in MRI scanners per capita, access is generally not a concern, and studies have shown that patients typically have a relatively short wait time to receive treatment [63,64]. In European countries, however, the estimated wait time for the initiation of drug treatment following initial consultations can be up to one year long, which could be due (in part) to an overload of patients seeking facilities with the appropriate MRI technology [65]. Access to MRI scanners, in terms of both availability and cost, remains an issue for drugs like lecanemab or donanemab.

Further concerns remain from the treatment centres’ perspectives, as (in the case of Japan) outpatient space and staffing for treatments (both for administration of drugs and follow-up MRI) could be insufficient for the scale of the problem faced with AD [64]. To address the issue of limited facility space, new drug administration methods are being explored. In the United States, subcutaneous administration of lecanemab has been approved (lecanemab-irmb), and this treatment can be administered by oneself, in the comfort of the person’s home (with a subcutaneous autoinjector) [66]. Some early research involving subcutaneous injections of lecanemab has shown comparable results (of Aꞵ removal and pharmacokinetics) to IV injection, with fewer infusion-related reactions and the potential for a lower risk of ARIA-E [67].

Concerning drug delivery and action within the body, issues with crossing the BBB remain. To address this, drug delivery mechanisms like lipid nanoparticles (LNPs), adeno-associated virus (AAV) vectors, and even ultrasound disruption of the BBB are being investigated [56]. For amyloid-focused therapies, a recent study has emerged that explores the low-density lipoprotein receptor-related protein 1 (LRP1) and its role in Aβ clearance [68]. By using LRP1-targeted polymersomes, the study found that receptor-mediated transport of Aꞵ could be modulated and upregulated, which, in AD model mice, reduced brain Aꞵ levels by nearly 45% [68]. Additionally, new monoclonal antibodies, such as trontinemab and remternetug are in clinical and Phase III trials, respectively [62]. Trontinemab, which features a Brainshuttle^TM^ technology and avoids binding to soluble Aβ, has enhanced delivery across the BBB, and early research has shown it to achieve comparable Aβ clearance with a lower risk of peripheral effects and ARIA-E [69]. Alternatively, remternetug has been put forward following donanemab, and is administered via subcutaneous injections that target aggregated forms of Aꞵ in plaques [62].

Another major shift is the conduct of trials for AD prevention and precision medicine. As it stands, the timing of intervention is often too late—when clinical symptoms emerge, neurodegeneration is already happening (synaptic loss, glial dysfunction, tau pathology, etc.). Efforts are being made to treat early-stage or even pre-symptomatic AD, with a focus on biomarkers that can be used to detect the preclinical disease. With amyloid-PET, tau-PET, CSF biomarkers, and other methods, the identification of individuals with pathological changes could be made years before clinical symptoms appear [62]. Additionally, the outcome measures for treatments are evolving—endpoints now integrate biomarkers, cognitive assessments, and functional measures, which more accurately demonstrate whether drugs have genuinely beneficial therapeutic benefits [56]. As a whole, shifts are moving towards a more personalized and stage-sensitive therapeutic model. If the right patients are selected at the right time and have the right markers/outcomes measured, then trials may just transform the development of drug treatments for AD for the better.

All of this does not come without limitations, unfortunately. Currently, issues with accessibility, such as high treatment costs and strict eligibility requirements, make it difficult for people to obtain the care they need, and pose problems to sponsors of said drugs. As of 2022 in the United States, aducanumab’s base price was $20,500 for the first year, with subsequent years costing $28,200—this is estimated not to be cost-effective by US standards, and it would only become so if priced below $3000 per year [70]. Comparably, as of 2024, lecanemab, priced at $26,500 annually, becomes cost-effective only at prices of $5100 per year or lower, and only when directed at patients meeting both genetic and symptomatic eligibility requirements [71]. With costs as high as they are, not only are treatments outside the reach of many, but a considerable market is also missed from companies’ perspectives, and it becomes cost-ineffective to produce these drugs.

As previously mentioned, meeting eligibility requirements for drug treatments also remains a hurdle for many patients. For instance, in order to be considered for lecanemab, individuals have to meet set lists of inclusion and exclusion criteria, the former including (but not limited to) the following: positive PET or CSF studies indicative of AD, diagnosis of MCI or mild AD, MMSE 22–30, and positive biomarkers for brain amyloid pathology [72]. Similar requirements, such as MCI or mild dementia consistent with AD Clinical Stages 3 or 4, an MMSE score of 20–30, and MRI evidence of ARIA status (specifically, fewer than 4 cerebral microbleeds), must be met for patients to be eligible for donanemab [73]. Strict adherence to these eligibility requirements, while capturing a specific portion of the population affected by AD, can miss out on those who could still benefit from treatment.

In conclusion, future of AD treatments are likely to rely on early detection, precision medicine, and multimodal interventions. Key priorities for the field include defining optimal treatment windows, refining biomarker-guided patient stratification, and determining how genetic factors such as *APOE* status should inform therapeutic selection and long-term management. Critical unanswered questions remain regarding the durability of treatment benefits, the integration of non–amyloid-based targets, and the development of combination therapies capable of addressing disease heterogeneity. Though many obstacles remain, especially in regard to cost-effectiveness and accessibility issues, recent and ongoing innovations in AD research mark a continued shift toward more personalized, mechanism-driven, equitable, and clinically meaningful approaches to prevailing over such a complex disease.

## Figures and Tables

**Table 1:** Summary of FDA-approved monoclonal antibody therapies.

Drug Name	FDA Approval Date	MoA	Administration	Adverse Effects	Benefits
Aducanumab	June 2021 (accelerated)	Fully human anti-Aβ IgGI	IV, every 4 weeks at a dose of 10 mg/kg	Phase III: ARIA-E 35.2% of patients on 10 mg/kg Sx: headache, confusion, dizziness, nausea. Double the percentage of ARIA-E in apolipoprotein E ε4 carriers compared to non-carriers	EMERGE: 22% reduction in decline in CDR-SB for high dose compared to placebo at week 78 Herring, et al., 2021 [28]: a lower lifetime probability of transitioning to institutionalization (25.2% vs. 29.4%)
Lecanemab	July 2023	Humanized IgGI with high affinity to Aβ soluble protofibrils	IV, biweekly at a dose of 10 mg/kg	Clarity: Infusion-related rxn (26.4%), ARIA-E (12.6%), 32.6% in homozygotes for apoE4, 10.9% of apoE4 carriers, 5.4% of non-carriers	Phase II: On CDR-SB, clinical decline was reduced by 26% at 10 mg/kg biweekly at 18 months Clarity: CDR-SB from baseline was 0.45 lower in those who received lecanemab, 1.21 as opposed to placebo, 1.66.
Donanemab	July 2024	Humanized IgGI which binds to Aβ	IV, once monthly 10 mg/kg	Phase Ia: More than 90% of patients developed antidrug antibodies TRAILBLAZER-ALZ 2: ARIA-E and -H occurred in 24% and 31.4% of participants treated with donanemab, with 6.1% experiencing symptomatic ARIA-E Homozygous and heterozygous apoE4 carriers experienced ARIA-E at a higher frequency than noncarriers, 40.6% and 22.8% as opposed to 15.7%	TRAILBLAZER-ALZ 2: 36% reduction in decrease in CDR-SB over 18 months

## Data Availability

No new data were created or analyzed in this study. Data sharing is not applicable to this article.
